# Food Intake According to Clock Gene Polymorphisms: A Systematic Review

**DOI:** 10.1096/fj.202500951R

**Published:** 2025-08-11

**Authors:** Laís Teixeira Campos, Polimar Ferreira Fonseca, Daniela Mayumi Usuda Prado Rocha, Josefina Bressan, Ana Claudia Pelissari Kravchychyn, Helen Hermana Miranda Hermsdorff

**Affiliations:** ^1^ Laboratory of Clinical Analysis and Genomics; Laboratory of Energy Metabolism and Body Composition, Department of Nutrition and Health (DNS) Universidade Federal de Viçosa (UFV) Viçosa Minas Gerais Brazil

**Keywords:** circadian clocks, energy intake, genotype, mealtimes, single nucleotide polymorphism

## Abstract

This systematic review investigated differences in daily energy intake among genotypes of circadian clock genes, potentially supporting personalized nutritional strategies for health. This topic can help develop personalized nutritional strategies for metabolic health by evaluating SNPs in circadian clock genes that may influence dietary intake. We searched the PubMed, EMBASE, and Cochrane Library databases following PRISMA guidelines and assessed the risk of bias using the Joanna Briggs Institute (JBI) tool (PROSPERO: CRD42024601530). Ten studies were included, involving 12,115 adult and elderly participants (mean age 40.8 ± 8.6 years; 60.6% women). Six studies investigated the SNP *CLOCK* rs1801260, while four analyzed the SNPs *CLOCK* rs4580704, *CLOCK* rs3749474, *CRY1* rs2287161, and *CRY2* rs11605924, with one study for each. Only one study found an association between the minor allele of *CLOCK* rs1801260 and increased energy, carbohydrate, and lipid intake, as well as later meal timing. Individuals with at least one risk allele (C) had higher intake and later mealtimes than those with the wild‐type allele (TT). The inconsistent associations across studies may be attributed to methodological limitations, including dietary assessment, sample size, genetic model classification, population characteristics, and environmental factors, such as including dietary patterns, chronotype, night shift work, sleep, and meal timing. Future research should adopt more comprehensive approaches to better clarify the impact of circadian gene variants on eating behavior.

## Graphical abstract

The study used PubMed, Embase, and Cochrane databases, screening with Rayyan QCRY. Ten studies were included, totaling 12 115 adults (40.8 ± 8.6 years, mostly women). SNPs analyzed were CLOCK (rs1801260, rs4580704, rs3749474) and Cry1/Cry2 (rs2287161, rs11605924). No studies examined Bmal, PER, Rev‐erb, or ROR SNPs. Only Rahati et al. found a significant link between CLOCK rs1801260 and the primary and secondary aims. Methodological and environmental limitations may explain inconsistent associations.

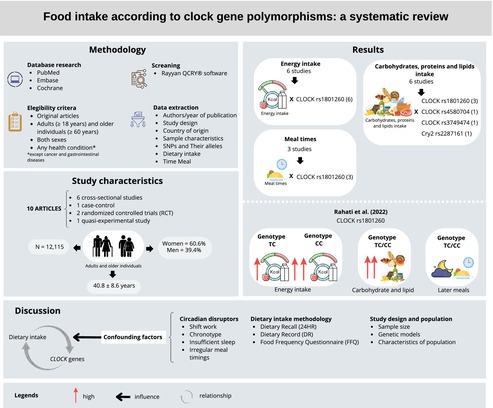

## 1 Introduction

The circadian clock regulates behavioral and mood rhythms, as well as cellular processes and physiological functions, throughout the light–dark cycle [1, 2]. The circadian rhythm, which follows a 24‐h cycle, is the most extensively studied and influences a wide range of biological functions [1, 2]. This rhythm synchronizes the organism with its environment to maintain physiological processes regulated over time [1, 3, 4]. The circadian clock is understood as central, located in the hypothalamus and regulated by the light–dark cycle in the suprachiasmatic nuclei (SCN), and peripheral, present in other tissues such as the liver, muscles, adipose tissue, and intestine [1, 4, 5].

The transcription of clock genes regulates the circadian rhythm at the cellular level, including clock circadian regulator (*CLOCK*), basic helix–loop–helix ARNT like 1 (*Bmal1*), basic helix–loop–helix ARNT like 2 (*Bmal2*), period circadian regulator 1 (*PER1*), period circadian regulator 2 (*PER2*), period circadian regulator 3 (*PER3*), cryptochrome circadian regulator 1 (*CRY1*) and cryptochrome circadian regulator 2 (*CRY2*). At the molecular level, there is a central cycle in which positive transcriptional activators such as CLOCK and BMAL1 form a heterodimer (CLOCK:BMAL1), which induces the expression of the *PER1*/*2*/*3* and *CRY1*/*2* genes by binding to specific DNA sequences known as E‐boxes. The PER and CRY proteins accumulate in the cytoplasm and inhibit CLOCK:BMAL activity through negative feedback, without affecting transcription. Once PER and CRY are degraded, the cycle resumes, maintaining a 24‐h rhythm [5, 6]. The CLOCK:BMAL1 heterodimer is also involved in an accessory loop that is interconnected with the core cycle, inducing the expression of nuclear receptors, such as nuclear receptor subfamily 1 group D member 1 (*NR1D1*), also know as *Rev‐erbα* and RAR related orphan receptor A (*RORA*), also known *Rorα* [5, 6, 7, 8]. These receptors regulate the transcription of *Bmal1*, with REV‐ERBα acting as a repressor and RORα as an activator [5, 7, 8]. Together, they form a secondary feedback loop that reinforces and stabilizes the core E‐box‐mediated cycle [9].

The circadian misalignment, induced by factors such as inadequate exposure to sunlight, irregular sleep patterns, or unhealthy food intake, negatively affects factors involved in energy balance and weight control. At the same time, disruptions in light–dark and feeding/fasting cycles appear to promote metabolic patterns related to obesity [10]. Furthermore, circadian rhythm disturbances have been associated with an increased risk of cardiometabolic diseases, including impaired glycemic control, type 2 diabetes, and obesity [4, 11]. In this context, the deletion of the *Bmal2* gene (a paralog of *Bmal1*) can lead to metabolic alterations, such as mild obesity, changes in energy and food metabolism patterns, and circadian changes, including the loss of rhythmicity of metabolic genes in brain clocks [12]. Additionally, studies with adipose tissue‐specific *Bmal1* knockout mice and *PER* and *CRY* mutants showed inhibition of BMAL1 production, which was correlated with suppressed leptin expression in adipose tissue [13]. Similarly, a systematic review compiled evidence from animal studies indicating that multi‐tissue *Clock* mutant mice exhibit reduced gene expression and disrupted circadian regulation [14] [15]). These alterations result in a phenotype characterized by increased food intake and obesity, with these effects being modulated by genetic background [15]. Moreover, genetic disruptions in the clock network, such as mutations, can affect glucose homeostasis, partly due to impaired insulin secretion and defects in pancreatic islet proliferation, as well as hypertriglyceridemia, possibly resulting from increased intestinal lipid absorption and hepatic overproduction [15].

In turn, Single Nucleotide Polymorphism (SNP) refers to variations in single base pairs of genomic DNA that naturally occur in normal individuals [16]. These polymorphisms can occur in coding regions or regulatory functions, but are most often found in intergenic regions with no determined function. Therefore, not all SNPs have a phenotypic impact [17]. Polymorphisms in the *CLOCK* gene, specifically the C allele of SNP rs1801260, have been associated with shorter sleep duration and delayed sleep onset in patients with depression and bipolar disorder [18]. SNPs in genes such as *PER2*, *CRY1*, *REV‐ERBα*, and *CLOCK* have also been linked to metabolic disorders [19]. The *PER2* SNP rs2304672C>G has been associated with abdominal obesity, with its minor allele linked to a higher likelihood of excessive snacking, diet‐related stress, skipping breakfast, and abandoning dietary treatment for obesity compared to non‐carriers [20]. The SNP rs3749474 of the *CLOCK* gene is associated with a higher BMI compared to non‐carriers. Additionally, carriers of the minor allele exhibit higher fat intake and a significantly increased risk of overweight or obesity [21, 22].

The development of a systematic review with this focus is justified by the need to compile scientific literature data on the influence of key SNPs in circadian CLOCK genes on food consumption. Considering the increasing prevalence of obesity and the changes in sleep patterns, studying the relationship between two factors influencing these conditions—SNPs in circadian CLOCK genes and food consumption—may provide opportunities for personalized nutritional strategies aimed at metabolic health by integrating principles of precision nutrition [10, 23, 24, 25].

This review aims to investigate the effect of SNPs in *CLOCK*, *1/2*, *PER*, *CRY*, *Rev‐erb*, and *ROR* genes on daily energy intake in adults. As secondary objectives, the review analyzes whether these SNPs influence macronutrient intake or meal timing. The findings may contribute to the development of personalized dietary approaches, considering each patient's circadian individuality and how their food consumption responds to these factors.

## 2 Materials and Methods

This systematic review was conducted following the Preferred Reporting Items for Systematic Reviews and Meta‐Analyses (PRISMA) guidelines [26]. The review protocol was registered in the International Prospective Register of Systematic Reviews (PROSPERO: CRD42024601530). The research was based on the components of the PECO acronym (P = Population; E = Exposure; C = Comparison; O = Outcome), guided by the question: “Is food consumption different between individuals with the risk allele or the wild‐type allele of circadian clock genes SNPs?” (Table S1). As secondary outcomes, we looked for differences in macronutrient intake and meal timing between the genotypes. Due to the type of study, ethical approval was not required under local legislation.

### 2.1 Search Strategy

Three electronic databases were used for the bibliographic search: PubMed, Embase, and Cochrane Library. The search was conducted in October 2024 in duplicate, without applying filters or language restrictions. Controlled descriptors were selected based on Medical Subject Headings (MeSH), Descriptors in Health Sciences (DeCS), and Emtree terms in English. Additionally, free terms related to the topic were included. Boolean operators OR and AND were used to connect terms during the search.

The search strategy included the terms “Single Nucleotide Polymorphisms” AND (Clock OR Bmal OR Per OR Rev‐erba OR Ror OR “Circadian Rhythm”) AND (“energy intake” OR carbohydrate OR protein OR lipid), along with their synonyms, abbreviations, and plurals according to each database (Table S2).

### 2.2 Inclusion and Exclusion Criteria

The inclusion criteria were original articles that evaluated humans and at least one SNP of circadian clock genes and energy intake. Participants included adults (≥ 18 years) and/or the elderly (≥ 60 years) of both sexes and any health condition, except cancer and gastrointestinal diseases. No restrictions were placed on article publication dates.

The exclusion criteria included reviews, book chapters, conference abstracts or proceedings, letters to the editor, publications without full‐text availability, animal studies, and manuscripts that did not meet the inclusion criteria.

### 2.3 Screening and Selection of Articles

The references identified were imported into the Rayyan QCRY software [27]. Using this tool, duplicates were removed, and two independent reviewers (P.F.F. and L.T.C.) manually screened titles and abstracts in parallel based on the inclusion and exclusion criteria. In cases of disagreement during the title and abstract screening process, the article was included in the full‐text review phase. Discrepancies identified during the full‐text evaluation, including challenges in retrieving necessary data and related uncertainties, were resolved through consensus between the two reviewers (P.F.F. and L.T.C.). Full‐text articles were accessed through institutional access via CAPES (https://www‐periodicos‐capes‐gov‐br.ez35.periodicos.capes.gov.br/).

### 2.4 Risk of Bias Assessment

All studies included in the systematic review were individually assessed for risk of bias by two researchers (P.F.F. and L.T.C.), independently and in parallel. Any discrepancies in the assessments, such as divergences in blinding domains, were resolved by consensus. The assessment was conducted using the Joanna Briggs Institute (JBI) critical appraisal tools for each type of study [28, 29]. Studies were classified as low (> 70%), moderate (50%–70%), or high risk of bias (< 50%) based on the percentage of affirmative responses [30].

### 2.5 Data Extraction

After selecting the studies, the aspects considered for the synthesis table were determined by consensus among the authors. Data extracted from each study were the authors and year of publication, study design, country of origin, sample characteristics (sample size, sex, and age), SNPs and their alleles, dietary intake (daily energy intake, carbohydrates, proteins, and lipids) and time of meal. Two authors (P.F.F and L.T.C) extracted the data and reviewed the information to ensure accuracy.

## 3 Results

### 3.1 Selection and Characteristics of the Studies

**FIGURE 1:**
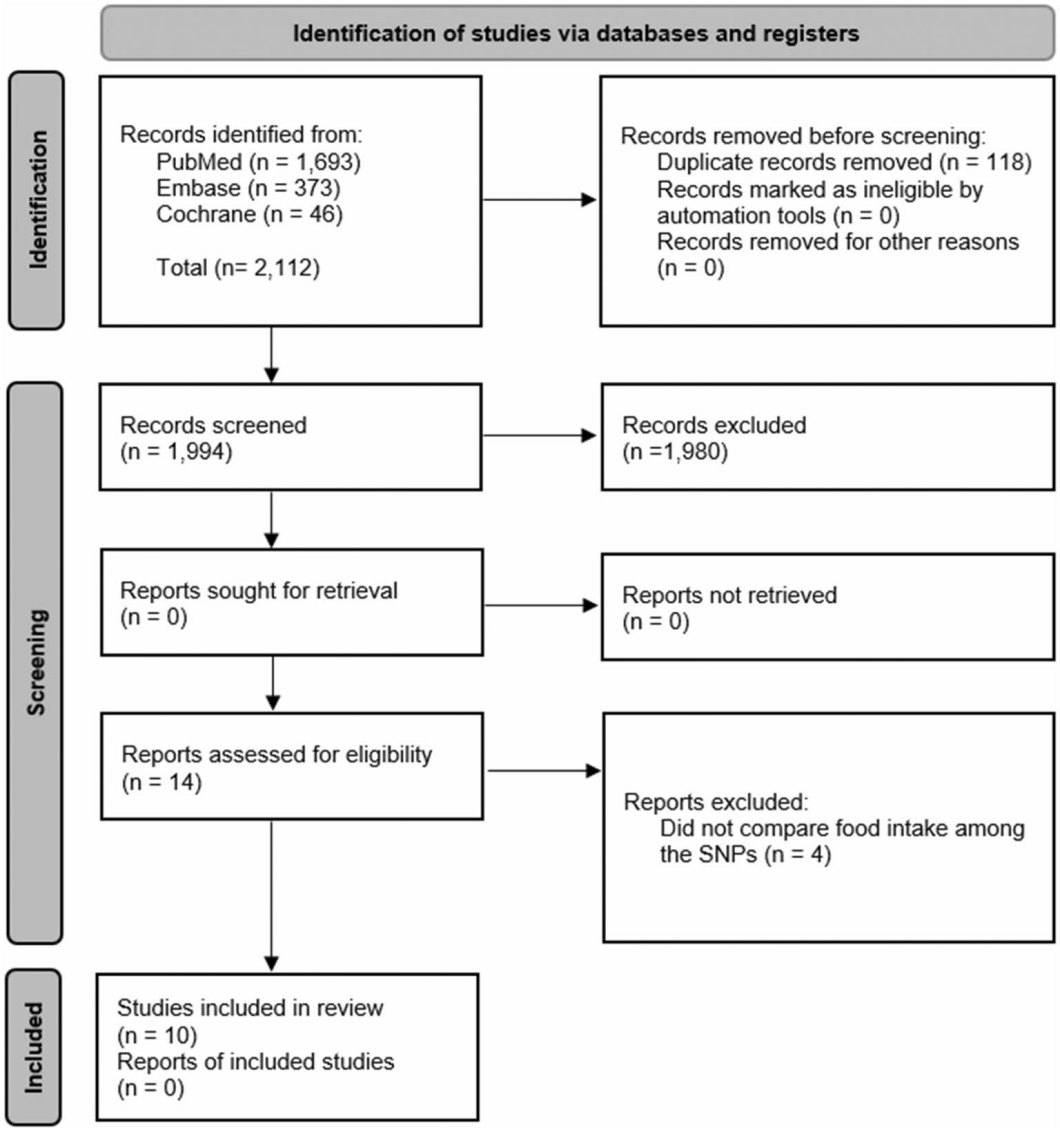
PRISMA flowchart of bibliographic search procedures and study selection [26].

A total of 2112 references were retrieved from the PubMed, Embase, and Cochrane Library databases. After duplicate removal, 1994 titles remained. During the screening of titles and abstracts, 1980 records were excluded based on the initial exclusion criteria. Of the 14 titles accepted for full‐text reading, four were excluded because they did not compare daily energy intake between the SNP alleles. At the end of the process, 10 articles were included in this systematic review (Figure 1).

This systematic review included six cross‐sectional studies, one case–control study, two randomized controlled trials (RCTs), and one quasi‐experimental study. Six of the articles were conducted in Spain, two in Iran, one in the United States, and one in Japan. The initial total sample consisted of 12 115 adults and elderly individuals, with an average age of 40.8 ± 8.6, predominantly women (60.6%; *n* = 7346). The SNPs of the *CLOCK* gene were studied, including rs1801260 [31, 32, 33, 34, 35, 36], rs4580704 [37], rs3749474 [38], as well as the *CRY1* and *CRY2* genes, respectively, rs2287161 [39] and rs11605924 [40]. No studies were found that evaluated daily energy for SNPs of the *Bmal1*/*2*, *PER*, *Rev*‐*erb*, and *ROR* genes (Table 1).

**TABLE 1:** Methodological characteristics of the studies.

References	Country	Study type	Number of participants			Years (mean ± SD)	Characteristics of participants	Method of collecting food consumption
			Total	Men	Women			
Garaulet et al. [34]	Spain	Quasi‐experimental	1495	262	1233	39.4 ± 12.3	Overweight or Obesity (BMI 25 a 40 kg/m^2^)	24‐h dietary recall
Bandín et al. [32]	Spain	Cross‐Sectional	85	—	85	43.0 ± 12.0	Overweight (BMI 25 a 29.9 kg/m^2^)	24‐h dietary recall
Galbete et al. [33]	Spain	Cross‐Sectional	903	657	246	69.0 ± 6.0	Elderly population in general	FFQ
Yamaguchi et al. [35]	Japan	Cross‐Sectional	95	—	95	19.6 ± 0.2	Without comorbidities	2‐day dietary records
Mirzaei et al. [40]	USA	RCT	721	280	441	51.0 ± 9.2	Overweight or Obesity (BMI 25 a 40 kg/m^2^)	5‐day dietary records
Corella et al. [37]	Spain	RCT	7098	3028	4070	67.0 ± 6.2	DM2 or 3 or more cardiovascular risk factors	FFQ
Murube et al. [38]	Spain	Cross‐Sectional	898	329	569	41.0 ± 12.0	General population without severe illness	3‐day dietary records
Mirzababaei et al. [39]	Iran	Cross‐Sectional	377	—	377	36.7 ± 9.1	Overweight or Obesity (BMI 25 a 45 kg/m^2^)	FFQ
Yang et al. [36]	Spain	Case–control	40	—	40	44.2 ± 10.5	Overweight or Obesity (BMI > 25 kg/m^2^)	7‐day dietary records
Rahati et al. [31]	Iran	Cross‐Sectional	403	213	190	36.5 ± 8.7	Overweight or Obesity (BMI 25 a 40 kg/m^2^)	7‐day dietary records

Abbreviations: BMI, Body Mass Index; DM2, Type 2 diabetes mellitus; FFQ, Semi‐quantitative food frequency questionnaire; RCT, Randomized Controlled Trials; SD, Standard Deviation; USA, United States of America.

### 3.2 Clock SNPs

Six studies investigated daily energy intake in relation to the *CLOCK* SNP rs1801260, and only one of them observed differences between the genotypes. Rahati et al. [31] identified higher daily energy intake in participants with at least one risk allele (TC), while those with a homozygous genotype (CC) showed the highest values. For the *CLOCK* rs4580704, *CLOCK* rs3749474, *CRY1* rs2287161, and *CRY2* rs11605924, only one study was found for each, and none indicated differences in daily energy intake between the genotypes (Table 2).

**TABLE 2:** Food intake and meal times according to clock gene polymorphisms.

References	Gene	SNP	Genotype	Food consumption				Meal times		
				Energy intake (kcal/d)	Carbohydrates (g/d)	Protein (g/d)	Lipids (g/d)	Breakfast (h ± min)	Lunch (h ± min)	Dinner (h ± min)
Garaulet et al. [34]	*CLOCK*	rs1801260	TT	2070 ± 35	151 ± 3	85 ± 2	87 ± 2	—	—	—
			CC + CT	2087 ± 38	146 ± 3	87 ± 2	96 ± 2	—	—	—
			*p*	0.741	0.149	0.257	0.894	—	—	—
Bandín et al. [32]	*CLOCK*	rs1801260	TT	1818 ± 578	—	—	—	8:48 ± 98	14:50 ± 33	21:26 ± 51
			TC + CC	2078 ± 771	—	—	—	9:24 ± 60	14:47 ± 30	21:27 ± 49
			*p*	0.486	—	—	—	0.130	0.670	0.580
Galbete et al. [33]	*CLOCK*	rs1801260	TT	2256 ± 660	254 ± 100	103 ± 38	85 ± 29	—	—	—
			TC + CC	2232 ± 637	245 ± 89	102 ± 28	86 ± 31	—	—	—
			*p*	0.581	0.180	0.811	0.414	—	—	—
Yamaguchi et al. [35]	*CLOCK*	rs1801260	TT	1582 ± 55	—	—	—	—	—	—
			TC + CC	1653 ± 55	—	—	—	—	—	—
			*p*	0.399	—	—	—	—	—	—
Yang et al. [36]	*CLOCK*	rs1801260	TT	1893 ± 601	—	—	—	8:35 ± 48	14:20 ± 23	21:25 ± 24
			TC + CC	1980 ± 767	—	—	—	9:30 ± 64	14:51 ± 29	21:32 ± 32
			*p*	NS	—	—	—	NS	NS	NS
Rahati et al. [31]	*CLOCK*	rs1801260	TT	2096 ± 437 ab	310 ± 80 a	83 ± 25	62 ± 18 ab	7:48 ± 54 ab	13:42 ± 42 ab	20:12 ± 48 ab
			TC	2232 ± 416 a	323 ± 66 b	82 ± 20	77 ± 24 a	8:36 ± 60 a	14:30 ± 54 a	21:00 ± 48 a
			CC	2434 ± 526 b	355 ± 92 ab	88 ± 25	84 ± 22 b	9:18 ± 60 b	15.06 ± 54 b	21:30 ± 48b
			*p*	< 0.001*	0.001*	0.180	< 0.001*	< 0.001*	< 0.001*	< 0.001*
Corella et al. [37]	*CLOCK*	rs4580704	CC	2275 ± 603	240 ± 81	92 ± 23	99 ± 30	—	—	—
			CG	2281 ± 605	239 ± 81	93 ± 23	99 ± 31	—	—	—
			GG	2272 ± 605	240 ± 81	93 ± 23	98 ± 30	—	—	—
			*p*	0.887	0.987	0.558	0.510	—	—	—
Murube et al. [38]	*CLOCK*	rs3749474	TT	2325 ± 508	214 ± 39	99 ± 17	106 ± 16	—	—	—
			TC	2202 ± 484	212 ± 33	97 ± 15	97 ± 14	—	—	—
			CC	2258 ± 466	224 ± 38	99 ± 16	101 ± 15	—	—	—
			*p*	0.159	0.305	0.717	0.250	—	—	—
Mirzaei et al. [40]	*Cry 2*	rs11605924	AA	1900 ± 509	—	—	—	—	—	—
			AC	2006 ± 586	—	—	—	—	—	—
			CC	1948 ± 548	—	—	—	—	—	—
			*p*	0.280	—	—	—	—	—	—
Mirzababaei et al. [39]	*Cry 1*	rs2287161	GG	2740 ± 828	392 ± 131	94 ± 32	95 ± 31	—	—	—
			GC + CC	2635 ± 798	372 ± 12	92 ± 31	98 ± 34	—	—	—
			*p*	0.270	0.170	0.610	0.540	—	—	—

*Note:* Values are presented as mean ± standard deviation. a, b: statistical difference between genotypes.

Abbreviations: NS, not significant; SNP, single nucleotide polymorphism.

* *p*‐value statistically significant.

Six studies evaluated carbohydrate, protein, and lipid intake according to clock SNPs. Three articles examined the *CLOCK* SNP rs1801260, while SNPs *CLOCK* rs4580704, *CLOCK* rs3749474, and *CRY2* rs2287161 were each analyzed in a single study. Rahati et al. [31] found that the presence of the risk allele of *CLOCK* SNP rs1801260 was associated with higher consumption of carbohydrates and lipids (Table 2).

Three studies investigated the relationship between meal timing and the *CLOCK* SNP rs1801260. Only the study by Rahati et al. [31] found that participants with at least one risk allele tended to have later meals (Table 2).

A meta‐analysis was deemed infeasible in this systematic review due to the significant heterogeneity among the included studies. There was variation in methodological design, study populations, and the tools used for dietary data collection. Moreover, not all SNPs were assessed across all studies, making it unfeasible to perform pooled effect estimates. Finally, the findings were inconsistent primarily and not replicable, with only one study reporting a statistically significant association with the outcomes of interest [26].

### 3.3 Risk of Bias

**FIGURE 2:**
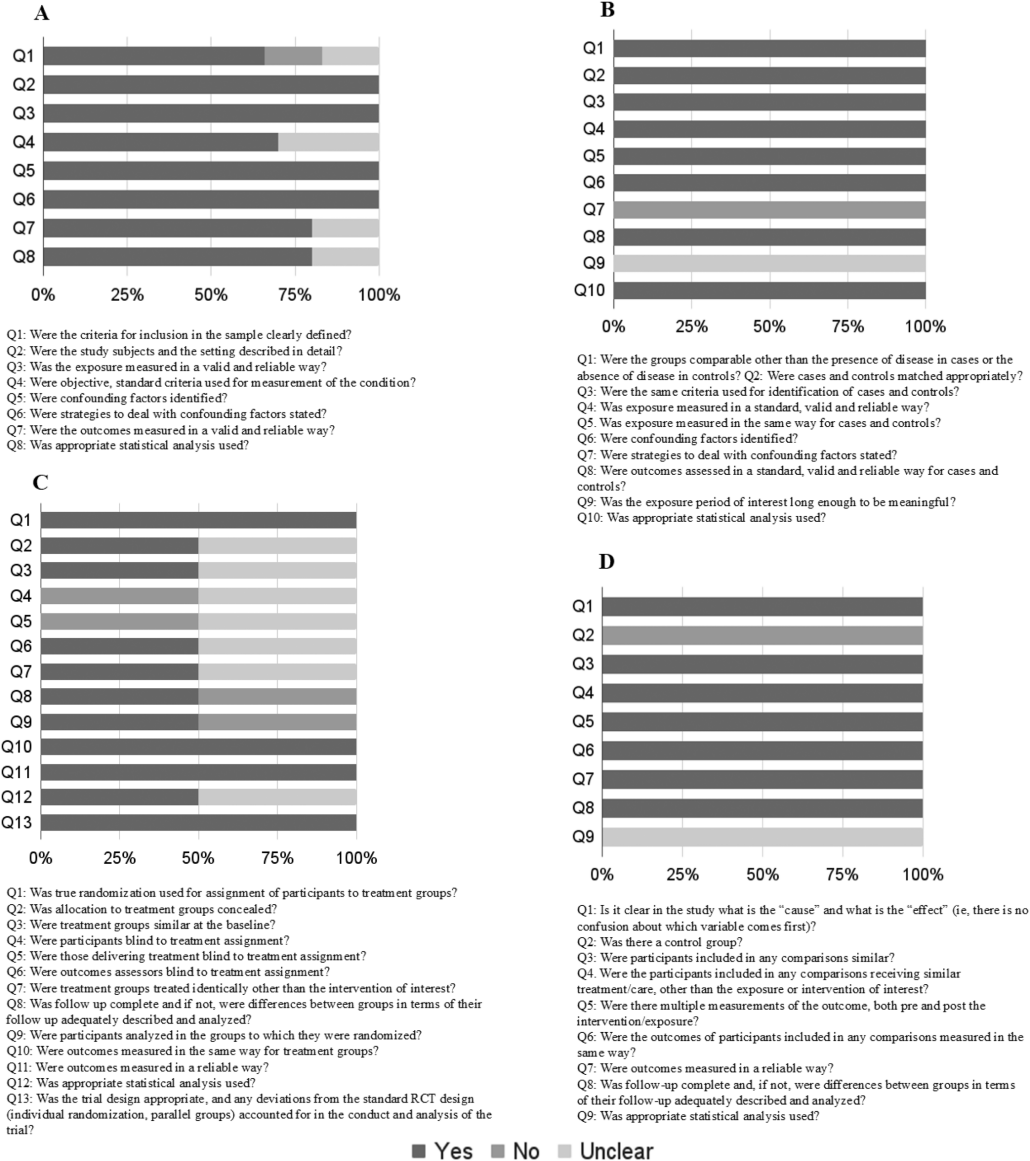
Risk of bias assessment for cross‐sectional (A), case–control (B), randomized controlled trials (C) and quasi‐experimental studies (D).

**FIGURE 3:**
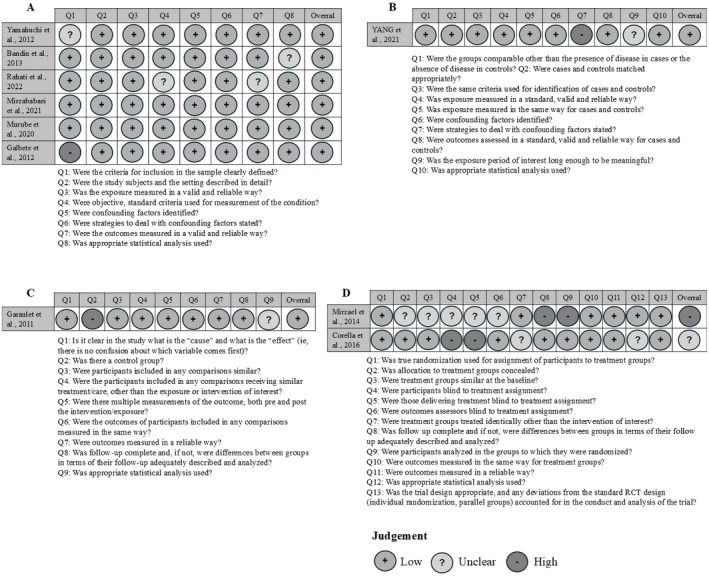
Risk of bias assessment of each study according to design: Cross‐sectional (A), case–control (B), quasi‐experimental studies (C) and randomized controlled trials (D).

Most studies provided sufficient information and had a low risk of bias (80%; *n* = 8). Only 10% (*n* = 1) of the studies showed a high risk of bias, and 10% showed a moderate risk of bias (*n* = 1). The risk of bias for each type of study is present in Figures 2 and 3.

Regarding cross‐sectional studies (*n* = 6), all domains exceeded a 60% acceptance rate. However, domain 1 had an article that did not present adequacy of the inclusion criteria and also had an article that was unclear on this subject. The domains on criteria and standardization to measure the study condition (domain 4), reliability and validity of the measurement of results (domain 7) and appropriate statistical analysis (domain 8) had articles that were unclear on this information.

As for the case–control study (*n* = 1), the identification of confounding factors (domain 7) was not presented, and it was unclear whether the exposure period of interest was sufficient to be significant (domain 9).

In the RCT studies (*n* = 2) 50% did not report on the blinding of participants or administrators of the treatments (domains 4 and 5, respectively), and 50% were unclear about these aspects. Concealed allocation of groups, similarity between treatment groups, blinding of evaluators, identical treatment (except for the intervention) in both groups, and appropriate statistical analysis (domains 2, 3, 6, 7 and 12 in that order) were unclear in 50% of the RCT studies. Complete group follow‐up and analysis according to randomization were not presented in 50% of the studies (domains 7 and 8). Domains 1, 10, 11, and 13 were fully reported in all articles.

The quasi‐experimental study (*n* = 1) did not meet the criterion regarding the existence of a control group (domain 2), and it was unclear whether the statistical analysis used was appropriate (domain 9).

## 4 Discussion

This systematic review explored differences in daily energy, macronutrient intake, and meal timings according to clock gene SNPs. Among the 10 studies included, six focused on the *CLOCK* SNP rs1801260, while the *CLOCK* SNPs rs4580704, *CLOCK* rs3749474, *CRY1* rs2287161, and *CRY2* rs11605924 were each analyzed in a single study each Only one study reported significant findings, linking the risk allele (C) of *CLOCK* SNP rs1801260 to higher energy, carbohydrates, and lipids intake, as well as later meal timing compared to the wild‐type allele (TT). No studies evaluated daily energy intake in relation to SNPs of the *BMAL1*/*2*, *PER*, *Rev*‐*erb*, and *ROR* genes.

The association between dietary intake and *CLOCK* gene variants was identified in only one study, with the findings likely influenced by unassessed confounding factors. Circadian disruptors, such as shift work, chronotype, insufficient sleep, and irregular meal timings, affect biological processes and disrupt the synchronization between central, peripheral, and molecular clocks [41]. The central clock regulates peripheral clocks located in various organs of the body. This system relies on transcription‐translation feedback loops, known as molecular clocks, to maintain cellular function [5]. Based on this framework, we hypothesize that the influence of SNPs may be amplified in conditions of pronounced circadian disruption.

The study by Rahati et al. [31] identified that individuals with the minor allele of the SNP rs1801260 in the *CLOCK* gene exhibited higher energy and macronutrient intake. Franzago et al. [10] demonstrated that this SNP is associated with various changes, including reduced sleep, altered ghrelin levels, nighttime food preferences, increased susceptibility to obesity, and difficulty with weight restriction. Garaulet et al. [21] have observed that carriers of the minor allele are more resistant to weight loss and metabolic changes induced by restrictive diets, as well as having higher BMI. These findings reinforce how the SNP rs1801260 is linked to metabolic alterations, eating behavior, and weight gain—factors that can directly influence dietary intake.

Animal model studies also provide important insights into how the *CLOCK* gene influences food intake. *CLOCK* gene mutant animals have shown alterations in the normal pattern of daytime feeding and developed hyperphagia and, consequently, obesity [42]. Mutations in the *CLOCK* gene can also affect macronutrient absorption. Pan and Hussain [43] observed that *CLOCK* mutant mice exhibited increased intestinal absorption of lipids and monosaccharides and decreased peptide absorption when compared to wild‐type mice. These findings suggest that the *CLOCK* gene plays a role not only in regulating feeding behavior, but also in intestinal absorption mechanisms. However, further studies are needed to understand how these effects manifest in humans.

Shift work is defined as employment outside conventional working hours and involves exposure to light at biologically inappropriate times [41, 44, 45]. This constitutes a pronounced form of circadian disruption and is particularly relevant in studies on biological rhythms [41, 45]. Nighttime light exposure can misalign cortisol, body temperature, and melatonin rhythms [45]. Regarding diet, night shift workers often alter meal timings, redistribute energy intake over the 24‐h period, increase snacking, and choose less healthy foods compared to daytime workers [41, 44, 46]. For example, in one study, nurses working night shifts showed higher intake of fats, sweets, and cereals, and lower consumption of fiber‐rich foods [47]. These dietary changes affect the hormonal regulation of appetite, increase meal skipping, and elevate the risk of cardiometabolic diseases [41]. Therefore, in addition to shift work itself, these variables should be carefully considered in research on circadian clocks and their health implications. Given its influence on eating patterns and metabolic regulation, shift work represents an important variable in studies focused on dietary intake. The relationship between shift work and phase delays in circadian clock genes, which may influence food intake, remains inconclusive. While some studies demonstrate this association, others do not, with discrepancies attributed to differences in study duration, length of night shift exposure, and measurement methodologies [45].

Chronotype is a marker of circadian phase that classifies individuals based on their preferences for activity and rest timing [48], and is categorized as morning, intermediate, or evening type [49]. In evening chronotypes, exposure to light at inappropriate times—especially in the early evening—can cause circadian disruptions due to phase delays [41, 46]. Furthermore, individuals with this chronotype tend to have poorer dietary quality, with higher energy density, lower physical activity levels, and greater cardiovascular risk compared to morning and intermediate types [41]. For instance, individuals with an evening chronotype often eat later, which may interfere with peripheral clocks in the liver and pancreas, negatively impacting optimal metabolism [41, 46]. In this context, a genome‐wide association study on chronotypes suggested that allelic variants in or near the *PER1*, *PER2*, *PER3*, and *CRY1* genes affecting the modification and regulation of the circadian clock contribute to population‐level variation in chronotype [50]. Moreover, other studies have shown that carriers of the 3111C allele or CC genotype of the *CLOCK* gene, as well as mutations in the *PER3* gene, exhibit evening preferences [51, 52]. Conversely, polymorphisms in the *PER1* and *PER2* genes have been associated with morning habits [53]. Therefore, chronotype and meal timing are essential factors to be considered in studies of food intake, particularly those investigating SNPs in circadian clock genes, since these elements also influence sleep and circadian phase.

The presence of polymorphisms in circadian clock genes can also impact sleep duration and sleep‐related characteristics. In an animal model study, the deletion of *Bmal1* in SCN VIP neurons resulted in reduced NREM sleep during the subjective light phase, leading to a redistribution of sleep–wake activity [54]. In a Japanese cohort study, the rs11113179 variant in *CRY1* was associated with an increased likelihood of sleep onset difficulties. Additionally, the rs1026071 and rs1562438 variants in *Bmal1* were also significantly associated with sleep initiation problems [55]. Furthermore, a Korean longitudinal study found that individuals carrying the C allele of SNP rs10002541, located in the *CLOCK* gene, exhibited a more pronounced reduction in sleep duration compared to individuals homozygous for the T allele. SNPs rs4580704 and rs6850524, also located in the same gene, were likewise associated with a greater decrease in sleep duration [56]. Haplotype analysis of the *CLOCK* gene showed that individuals carrying less common SNP combinations (GCCTC or CGTTC) had a significantly shorter sleep duration compared to those with the most common haplotype (CGTCT), suggesting a potential combined functional effect of these variants on sleep patterns [56]. Studies have shown that these alterations in sleep patterns can negatively affect diet quality by increasing the consumption of snacks and fats [57], as well as carbohydrates, sugars, and foods with a high glycemic index and load [58]. Yang et al. [59] observed that moderate sleep restriction increased hunger and food cravings, contributing to excessive energy intake. Therefore, understanding how specific polymorphisms in circadian clock genes influence sleep duration and quality is essential, as such alterations may trigger behavioral and metabolic changes that compromise dietary patterns.

An important circadian clock disruptor is meal timing, which was assessed in only three studies. Research indicates a secular trend toward later eating patterns [41, 60]. Late eating habits have been associated with adverse health outcomes, such as an increased risk of overweight/obesity, glucose intolerance, and reduced insulin secretion [61, 62]. A significant aspect related to meal timing is breakfast skipping, which was not evaluated in any of the studies reviewed. Skipping breakfast has been linked to poorer diet quality, such as lower intake of whole grains and fiber [63], and a higher risk of overweight and obesity [64]. Conversely, in the study by Gwin and Leidy [65], eating breakfast was associated with reduced appetite and food reward, increased satiety, and a decrease in unhealthy snack consumption compared to skipping breakfast.

In studies that found no association between SNPs and dietary intake, it is essential to consider factors that may have influenced these results. A well‐defined methodology is crucial to ensure reliable and validated findings [66]. Dietary assessment tools such as the 24‐h Dietary Recall (24HR), Dietary Record (DR), and Food Frequency Questionnaire (FFQ) are widely used but can present methodological variations [67]. The 24hHR is popular for providing detailed data but may lack precision due to reliance on the respondent's memory [67, 68]. The DR is collected over one or more days, preferably at the time of consumption, which can reduce memory bias but may introduce reactivity bias, altering food choices [67]. The FFQ, being simple and cost‐effective, quantifies intake based on frequency and portion size, offering a rough estimate of daily intake [67, 68]. However, its accuracy in energy quantification is generally inferior to that of the 24hHR and DR, making it recommended only when other methods are unavailable [67].

Moreover, achieving reliable results depends on a well‐trained interviewer and the use of strategies that facilitate the process, such as software and application methodologies like the Automated Multiple‐Pass Method (AMPM) for the 24hHR [67, 69]. The choice of method should also take into account the research objective, the relevance of dietary intake data, the time required for data collection, financial constraints, interview duration, and the characteristics of both the interviewer and the respondent [67, 70]. In this review, the FFQ was utilized in two studies: one randomized controlled trial (RCT) and one cross‐sectional study. In both cases, no statistically significant associations were found between the variables of interest and the outcomes reviewed.

Sample size is a critical factor for the validity and reliability of studies, particularly in epidemiological and genomic association research [71, 72]. Sample size calculations must account for alpha and beta errors, which represent the likelihood of false‐positive or false‐negative results, directly influencing the study's statistical power. Lower alpha and beta error rates enhance the robustness of conclusions [71, 73]. The prevalence of the disease and effect size are inversely related to sample size: lower values for these parameters necessitate a larger number of participants to achieve statistically significant results. Furthermore, higher allele frequencies, particularly for minor alleles, and stronger linkage disequilibrium reduce the sample size requirements [71, 73].

Genetic models (dominant, recessive, and additive) must be accurately specified to avoid reducing the study's statistical power. Classification errors, such as the misclassification of genotypes and phenotypes, can compromise the results but are challenging to quantify during the study design phase [71, 73]. Variants in circadian clock genes likely exert a modest effect on dietary intake, which may be influenced by polymorphism prevalence. This effect can be obscured if the sample size is insufficient or if the analyses lack robustness [71, 73, 74]. Therefore, sample size must be carefully planned and integrated with hypothesis development, study design, sampling techniques, and data analysis to ensure reliable outcomes [75].

Furthermore, the characteristics of the study population should be considered. Only three articles included populations without comorbidities, while the majority included individuals with comorbidities, especially overweight and obesity. Leptin resistance, commonly observed in individuals with obesity [76], is a relevant condition characterized by decreased responsiveness to leptin, even at high concentrations, leading to hyperleptinemia [77]. Leptin resistance can affect the regulation of food intake, nutrient absorption, metabolism, and insulin resistance, disrupting energy balance [77]. Other SNPs in genes related to food consumption and excess weight may also be present in the studied population, necessitating larger studies that account for these confounding factors [78]. Therefore, when evaluating food intake, it is essential to consider pathological and intrinsic factors in the participants that may influence the results.

One of the strengths of this review is the compilation of articles from the literature investigating CLOCK genes and their relationship with dietary intake. Additionally, it discusses the key aspects that should be considered in genomic association studies involving SNPs of circadian clock genes and food consumption.

On the other hand, it also has limitations. No studies were found evaluating the relationship between food consumption and the SNPs of the *Bmal1*/*2*, *PER*, *Rev*‐*erb*, and *ROR* genes, which limits the scope of the conclusions of this review, as these genes play essential roles in regulating the circadian clock and may also influence eating behavior and metabolism. Finally, the review is based on a population that already has significant metabolic conditions related to food consumption. Therefore, it may not capture the full complexity of the genetic interactions that regulate eating patterns, leaving out potentially relevant variants.

This review identified only one study associating SNPs of circadian CLOCK genes to daily energy, macronutrient intake, and meal timing, while SNPs of the B*mal1*/*2*, *PER*, *Rev‐erb*, and *ROR* genes and their relation to food intake have not been explored yet. The findings suggest that the absence of consistent associations between circadian CLOCK gene SNPs and dietary behaviors may be attributed to methodological and environmental limitations. Future research should adopt more comprehensive approaches incorporating both qualitative and quantitative analyses of eating patterns, as well as factors like night shift work, chronotype, sleep quality, and meal timing. Such methodologies are essential to better understand the role of circadian gene variants in eating behavior.

## Author Contributions

L. T. C. and P. F. F. conceived and developed the research and contributed to writing the manuscript. D. M. U. P. R. assisted in study methodology and designing the search strategy. A. C. P. K., H. H. M. H., and J. B. contributed throughout the manuscript development process with input on methodology, result presentation, and discussion development. All authors were involved in reviewing the manuscript.

## Conflicts of Interest

The authors declare no conflicts of interest.

## Supporting information


**Table S1:** fsb270913‐sup‐0001‐TableS1.docx.
**Table S2:** fsb270913‐sup‐0001‐TableS1.docx.

## Data Availability

Data sharing is not applicable to this article as no datasets were generated or analyzed during the current study.
