# Supplementary material for: Food Intake According to Clock Gene Polymorphisms: A Systematic Review
Source: FASEB J. 2025 Aug 11;39(15):e70913. doi: 10.1096/fj.202500951R (PMC12337243; doi:10.1096/fj.202500951R)
Supplement: Supplementary file 1 — Table S1: fsb270913‐sup‐0001‐TableS1.docx. Table S2: fsb270913‐sup‐0001‐TableS1.docx. [file FSB2-39-e70913-s001.docx]

**MATERIAL SUPPLEMENTARY**

**Supplementary Table S1 - PECO**

Research question: Is food consumption different between individuals with the risk allele or the wild-type allele of circadian clock genes SNPs?

| **P** | Adults and elderly individuals of both sexes |
| --- | --- |
| **I / E** | Risk allele of the genes: *CLOCK*, PER, CRY, Bmal, Rev-Erb E ROR |
| **C** | Wild-type allele of the genes: *CLOCK*, PER, CRY, Bmal, Rev-Erb E ROR |
| **O** | Primary: calories (kcal)  Secondary: macronutrients (carbohydrates, proteins, and lipids) and meal timing |
| **S** | Cross-sectional, cohort, case-control, and randomized controlled trial |

**Supplementary Table S2 - Search Strategy**

| **Database** |  | **Descriptors** | **Number of articles found** | **Date** |
| --- | --- | --- | --- | --- |
| **PubMed** | **#1** | "Polymorphism, Single Nucleotide"[MeSH Terms] OR "Polymorphism, Single Nucleotide" OR "Polymorphisms, Single Nucleotide" OR "Single Nucleotide Polymorphism" OR "Single Nucleotide Polymorphisms" OR "Single-Nucleotide Polymorphism" OR "Single-Nucleotide Polymorphisms" OR SNP OR SNPs | 210.874 | 25/10/24 |
|  | **#2** | "Circadian Clocks"[MeSH Terms] OR "Circadian Clocks" OR "Clock, Circadian" OR "Clocks, Circadian" OR clock OR "Bmal-1" OR "BMAL1" OR "BMAL 1" OR "Bmal1" OR "BMAL2" OR Bmal2 OR Cry1 OR Cry2 OR Cry3 OR CRY 1 OR CRY 2 OR CRY 3 OR Cryptochrome OR Cryptochrome 1 OR Cryptochrome 2 OR Cryptochrome 3 OR BMAL OR Bmal OR "Brain and Muscle ARNT-Like" OR Per1 OR Per2 OR Per3 OR Period 1 OR Period 2 OR Period 3 OR Rev-erba OR Rev-ErbA OR Ror OR ROR  OR "Circadian Rhythm"[MeSH Terms] OR "Circadian Rhythm" OR "circadian clock" OR "circadian cycle" OR "circadian fluctuation" OR "circadian periodicity" OR "circadian rhythmicity" OR "circadian variation" OR "day night rhythm" OR "ARNTL Transcription Factors" OR "ARNTL Transcription Factors" OR "aryl hydrocarbon receptor nuclear translocator-like" OR "arntl" OR "circadian locomotor output cycles kaput" OR cryptochromes OR "retinoic acid receptor-related orphan receptors" OR "Cryptochrome1" OR "Cryptochrome2" OR "Cryptochrome3" OR "Brain and muscle arnt-like" OR "PER 1" OR "PER 2" OR "PER 3" OR "food intake" OR "Retinoid-Related Orphan" OR "ARNTL Transcription Factors"[MeSH Terms] | 1.977,086 | 25/10/24 |
|  | **#3** | "Energy Intake"[MeSH Terms] OR "Energy Intake" OR "Caloric Intake" OR "Calorie Intake" OR "dietary energy" OR "energy intakes" OR "caloric intakes" OR "calorie intakes" OR "calorie intakes" OR "calory intake" OR “eating” [MeSH Terms] OR "food intake" OR "food intakes" OR "dietary intake" OR "dietary intakes" OR "Carbohydrates" OR "Proteins" OR "Lipids" OR "Dietary Carbohydrates"[MeSH Terms] OR "Dietary Fats"[MeSH Terms] OR "Dietary Proteins"[MeSH Terms] OR "food consumption" OR "food ingestion" OR "food uptake" OR "meal ingestion" OR "intake, caloric" OR "carbohydrate intake" OR "alimentary carbohydrate" OR "carbohydrate consumption" OR "carbohydrate consumption" OR "carbohydrate intake" OR "diet carbohydrate" OR "dietary carbohydrate" OR "dietary carbohydrate intake" OR "protein intake" OR "diet protein" OR "dietary protein" OR "food protein" OR "protein consumption" OR "protein feeding" OR "protein food" OR "protein intake" OR "protein nutrition" OR "fat intake" OR "alimentary fat" OR "diet fat" OR "diet fats" OR "dietary fat" OR "dietary fats" OR "dietary fatty acid" OR "dietary lipid" OR "fat consumption" OR "fat feeding" OR "fat ingestion" OR "fat intake" OR "fat, dietary" OR "fatty acid intake" OR "lipid intake" OR "nutrition, fat" OR "nutritional intake" OR "dietary Proteins" OR "dietary fats" OR "Dietary Fat" OR "dietary carbohydrates" OR "dietary carbohydrate" | 4.014.227 | 25/10/24 |
|  |  | **Total: #1 AND #2 AND #3** | 1.693 | 25/10/24 |
| **Embase** | **#1** | 'single nucleotide polymorphism'/exp OR 'polymorphism, single nucleotide' OR 'single nucleotide polymorphism' OR 'snp' OR 'snps' OR 'polymorphisms, single nucleotide' OR 'single nucleotide polymorphisms' OR 'single nucleotide polymorphisms'/exp | 316.325 | 25/10/24 |
|  | **#2** | 'circadian rhythm'/exp OR 'circadian clock' OR 'circadian clocks' OR 'circadian cycle' OR 'circadian periodicity' OR 'circadian rhythm' OR 'circadian rhythmicity' OR 'circadian variation' OR 'day night rhythm' OR 'rhythm, circadian' OR 'clock, circadian' OR 'clocks, circadian' OR 'clock' OR 'circadian locomotor output cycles kaput' OR 'bmal' OR 'brain and muscle arnt-like' OR 'bmal-1' OR 'bmal1' OR 'bmal 1' OR 'bmal-2' OR 'bmal2' OR 'bmal 2' OR 'cryptochrome'/exp OR 'cryptochrome' OR 'cryptochromes' OR 'cry1' OR 'cry2' OR 'cry3' OR 'cry 1' OR 'cry 2' OR 'cry 3' OR 'cryptochrome 1'/exp OR 'cryptochrome 1' OR 'cryptochrome1' OR 'cryptochrome 2'/exp OR 'cryptochrome 2' OR 'cryptochrome2' OR 'cryptochrome 3' OR 'cryptochrome3' OR 'per1' OR 'per2' OR 'per3' OR 'per 1' OR 'per 2' OR 'per 3' OR 'period 1' OR 'period 2' OR 'period 3' OR 'rev-erba' OR 'rev erba' OR 'retinoid-related orphan' | 228.694 | 25/10/24 |
|  | **#3** | 'food intake'/exp OR 'food consumption' OR 'food ingestion' OR 'food intake' OR 'food uptake' OR 'meal ingestion' OR 'food intakes' OR 'caloric intake'/exp OR 'caloric intake' OR 'dietary energy' OR 'energy intake' OR 'intake, caloric' OR 'intake, energy' OR 'calorie intake' OR 'intake, calorie' OR 'energy intakes' OR 'caloric intakes' OR 'calorie intakes' OR 'calory intake' OR 'eating'/exp OR 'eating' OR 'dietary intake'/exp OR 'dietary intake' OR 'nutritional intake' OR 'dietary intakes' OR 'carbohydrate intake'/exp OR 'alimentary carbohydrate' OR 'carbohydrate consumption' OR 'carbohydrate feeding' OR 'carbohydrate intake' OR 'diet carbohydrate' OR 'dietary carbohydrate' OR 'dietary carbohydrate intake' OR 'dietary carbohydrates' OR 'carbohydrates' OR 'protein intake'/exp OR 'diet protein' OR 'dietary protein' OR 'dietary proteins' OR 'food protein' OR 'intake, protein' OR 'protein consumption' OR 'protein feeding' OR 'protein food' OR 'protein intake' OR 'protein nutrition' OR 'proteins' OR 'fat intake'/exp OR 'alimentary fat' OR 'diet fat' OR 'diet fats' OR 'dietary fat' OR 'dietary fats' OR 'dietary fatty acid' OR 'dietary lipid' OR 'fat consumption' OR 'fat feeding' OR 'fat ingestion' OR 'fat intake' OR 'fat, dietary' OR 'fatty acid intake' OR 'ingestion, fat' OR 'lipid intake' OR 'nutrition, fat' | 2.934.979 | 25/10/24 |
|  |  | **Total: #1 AND #2 AND #3** | 373 | 25/10/24 |
| **Cochrane** | #1 | MeSH descriptor: [Polymorphism, Single Nucleotide] OR SNPs OR SNP OR "Single Nucleotide Polymorphism" OR "Single Nucleotide Polymorphisms" OR "Nucleotide Polymorphism, Single" OR "Nucleotide Polymorphisms, Single" OR  "Polymorphisms, Single Nucleotide" OR "Single-Nucleotide Polymorphism" OR "Single-Nucleotide Polymorphisms" | 5.650 | 29/10/24 |
|  | #2 | MeSH descriptor: [Circadian Clocks] OR "Systems, Circadian Clock" OR "System, Circadian Clock" OR "Clocks, Circadian" OR "Clock, Circadian" OR "Clock System, Circadian" OR "Circadian Clock Systems" OR "Clock Systems, Circadian" OR "Circadian Clock System" OR "Circadian Clock" OR "ARNTL Transcription Factors" OR MeSH descriptor: [Circadian Rhythm] OR "Rhythm, Twenty-Four Hour" OR "Twenty Four Hour Rhythm" OR "Rhythms, Circadian" OR "Rhythms, Twenty-Four Hour" OR "Twenty-Four Hour Rhythm" OR "Circadian Rhythms" OR "Rhythm, Circadian" OR "Twenty-Four Hour" OR "circadian rhythm" OR MeSH descriptor: [circadian clocks] OR "circadian clock" OR "circadian cycle" OR "circadian periodicity" OR "circadian rhythm" OR "circadian rhythmicity" OR "circadian variation" OR "day night rhythm" OR "rhythm, circadian" OR "clock, circadian" OR "clocks, circadian" OR "clock" OR "circadian locomotor output cycles kaput" OR "bmal" OR "brain and muscle arnt-like" OR "bmal-1" OR "bmal1" OR "bmal 1" OR "bmal-2" OR "bmal2" OR "bmal 2" OR "cryptochrome" OR "cryptochrome" OR MeSH descriptor: [cryptochromes] OR "cry1" OR "cry2" OR "cry3" OR "cry 1" OR "cry 2" OR "cry 3" OR "cryptochrome 1" OR "cryptochrome 1" OR "cryptochrome1" OR "cryptochrome 2" OR "cryptochrome 2" OR "cryptochrome2" OR "cryptochrome 3" OR "cryptochrome3" OR "per1" OR "per2" OR "per3" OR "per 1" OR "per 2" OR "per 3" OR "period 1" OR "period 2" OR "period 3" OR "rev-erba" OR "rev erba" OR "retinoid-related orphan" | 21.530 | 29/10/24 |
|  | #3 | MeSH descriptor: [energy intake] OR "calorie Intake" OR "caloric Intake" OR "intake, calorie" OR "intake, energy" OR MeSH descriptor: [eating] OR "food intake" OR "intake, food" OR "intake, macronutrient" OR "macronutrient Intakes" OR "macronutrient Intake" OR ingestion OR "dietary Intakes" OR "intake, Dietary" OR "dietary intake" OR "nutrient intake" OR "intake, nutritional" OR "intake, nutrient" OR "nutritional intakes" OR "nutrient Intakes" OR "nutritional intake" OR "food consumption" OR "food ingestion" OR "food uptake" OR "meal ingestion" OR "food intakes" OR "dietary energy" OR "intake, caloric" OR "energy intakes" OR "caloric intakes" OR "calorie intakes" OR "calory intake" OR "nutritional intake" OR "dietary intakes" OR MeSH descriptor: [carbohydrates] OR carbohydrate OR "carbohydrate intake" OR "alimentary carbohydrate" OR "carbohydrate consumption" OR "carbohydrate feeding" OR "carbohydrate intake" OR "diet carbohydrate" OR "dietary carbohydrate" OR "dietary carbohydrate intake" OR "dietary carbohydrates" OR MeSH descriptor: [proteins] OR protein OR "protein intake" OR "diet protein" OR "dietary protein" OR "dietary proteins" OR "food protein" OR "intake, protein" OR "protein consumption" OR "protein feeding" OR "protein food" OR "protein intake" OR "protein nutrition" OR MeSH descriptor: [Lipids] OR "lipid" OR "fat intake" OR "alimentary fat" OR "diet fat" OR "diet fats" OR "dietary fat" OR "dietary fats" OR "dietary fatty acid" OR "dietary lipid" OR "fat consumption" OR "fat feeding" OR "fat ingestion" OR "fat intake" OR "fat, dietary" OR "fatty acid intake" OR "ingestion, fat" OR "lipid intake" OR "nutrition, fat" | 222.956 | 29/10/24 |
|  |  | **Total: #1 AND #2 AND #3** | 48 | 29/10/24 |
